# Nitric Oxide Synthesis Metabolites—As Potential Markers in Chronic Kidney Disease in Children

**DOI:** 10.3390/cimb44080242

**Published:** 2022-08-07

**Authors:** Joanna Piechowicz, Andrzej Gamian, Ositadima Chukwu, Dorota Polak-Jonkisz

**Affiliations:** 1 Department of Medical Biochemistry, Wroclaw Medical University, 50-367 Wroclaw, Poland; 2 Hirszfeld Institute of Immunology and Experimental Therapy, Polish Academy of Sciences, 50-367 Wroclaw, Poland; 3 Jan Mikulicz-Radecki University Clinical Hospital, 50-556 Wroclaw, Poland; 4 Department of Pediatric Nephrology, Wroclaw Medical University, 50-367 Wroclaw, Poland

**Keywords:** nitric oxide metabolites, asymmetric dimethylarginine, symmetric dimethylarginine, citrulline, chronic renal failure

## Abstract

Nitric oxide (NO) is an important signaling molecule for many physiological and pathological processes. Diseases associated with abnormal NO synthesis include cardiovascular diseases, insulin-dependent diabetes, or chronic kidney disease (CKD). The aim of the paper was to evaluate NO synthesis metabolites, i.e., asymmetric dimethylarginine (ADMA), symmetric dimethylarginine (SDMA), dimethylamine (DMA), arginine, citrulline in plasma of patients with different severity of CKD and to seek possible links between these parameters and the development of this disease. Forty-eight CKD children and thirty-three age-matched controls were examined. Patients were divided into groups depending on the CKD stages (Group II-stage II, Group III-stage III, Group IV-stage IV, and Group RRT children on dialysis). To determine the concentrations of the above-mentioned metabolites in plasma liquid chromatography-mass spectrometry was used. There were significant differences observed in levels of ADMA, SDMA, DMA, and citrulline between control vis CKD groups (*p* values ranging from <0.001 to 0.029). Plasma arginine concentration was also higher in CKD patients compared to the control group but statistically insignificant. ADMA levels in CKD children were statistically significantly higher in relation to particular stages of CKD (RRT vis II stage of CKD: *p* = 0.01; RRT vis III-IV stages of CKD: *p* < 0.046). Citrulline levels in CKD children were statistically significantly higher in RRT group vis control (*p* < 0.001). Children with CKD develop disturbances in most metabolites of NO synthesis. Dialysis children treated show the greatest disturbances of plasma ADMA and citrulline levels. ADMA seems to be a good indicator of the gradual progression of the CKD, which is proved by the negative correlation with eGFR.

## 1. Introduction

Nitric oxide (NO) is an important signaling molecule for many physiological and pathological processes. Diseases associated with abnormal NO synthesis include cardiovascular diseases, insulin-dependent diabetes, or chronic kidney disease (CKD) [1,2,3,4]. NO is mainly synthesized in the human organism by three isoforms of nitric oxide synthase (NOS): endothelial synthase, neuronal cell one, and inducible calcium ion-dependent synthase. All these NOS isoforms use arginine and molecular oxygen as substrates and require cofactors, i.e., reduced NADPH, flavin adenine dinucleotide (FAD), flavin mononucleotide (FMN), and 5,6,7,8-tetrahydrobiopterin-BH4. The reaction of nitric oxide synthesis consists in a two-step reaction oxidation of arginine to citrulline and NO [5].

**Figure 1:**
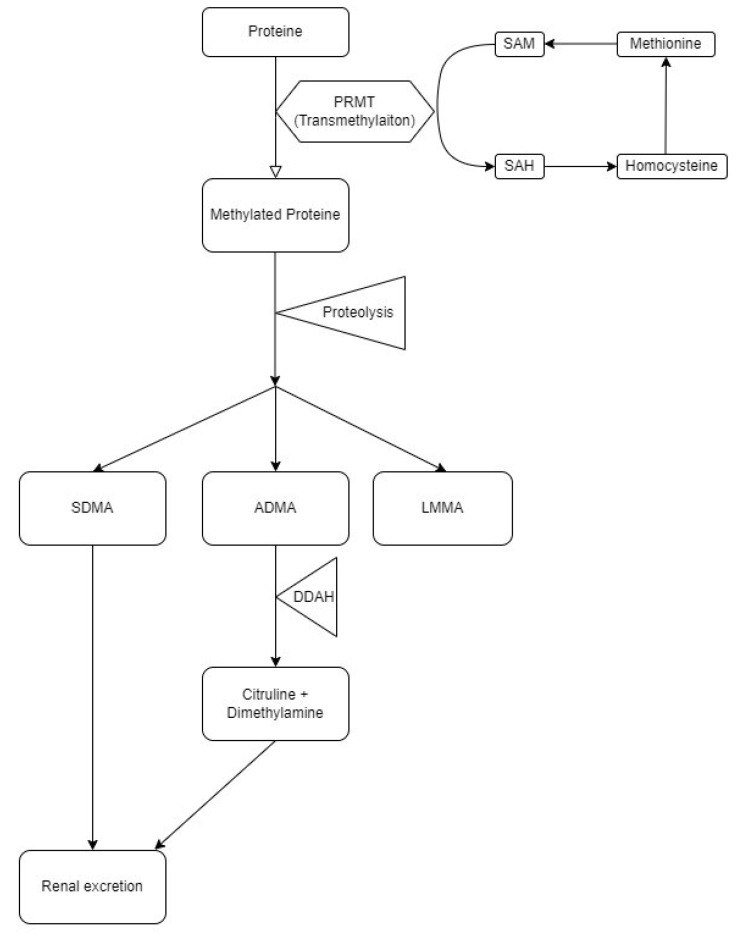
The diagram shows the metabolism of methylated arginine. PRMT protein arginine methyltransferases, SAM S-adenosylmethionine, SAH S-adenosylhomocysteine, SDMA symmetric dimethylarginine ADMA—asymmetric dimethylarginine, LMMA monomethyl-L-arginine, DDAH—Dimethylarginine dimethylaminohydrolase.

Arginine methylation leads to the production of asymmetric and symmetric dimethylarginine (ADMA and SDMA, respectively) (Figure 1). The synthesis of ADMA by S-adenosylmethionine (SAM) and degradation of ADMA is an actively regulated reaction. ADMA is a key inhibitor of NO synthesis, hindering both NOS activity and arginine cell uptake. It was found that SDMA did not have an inhibitory effect on NOS, but may indirectly interfere with NO synthesis by competing with arginine in transport by membrane cells. In a similar way to ADMA, competition of SDMA with arginine for cationic amino acid transporters responsible for transporting amino acids into the cell was found [6,7,8,9,10]. According to the above-mentioned researchers, the disturbed concentrations of arginine and products of its dimethylation may serve as a prognostic indicator of kidney function deterioration and CKD development [11].

Over the last 20 years, many studies have been conducted to verify the use of asymmetric dimethylarginine (ADMA) as a marker in CKD. High levels of ADMA have been reported, i.e., in cardiovascular diseases, hypercholesterolemia, neurogenic diseases, diabetes, and renal failure [12,13]. According to Ravani et al., in patients with chronic kidney disease, the serum ADMA levels are the third prognostic factor (immediately after hemoglobin concentrations and intensity of proteinuria) [14].

The concentration of structural isomer of endogenous nitric oxide synthase inhibitor, which is symmetrical dimethylarginine (SDMA), also correlates in plasma with GFR value; with the deterioration of renal function, an increase in its level is observed [15,16]. The concentration of SDMA in plasma does not depend on extra-renal factors, such as muscle mass, diet, inflammation, diabetes, or estrogen therapy. It has been observed that SDMA levels may be in the reference interval in diabetics, obese people, patients with liver disease, as well as those suffering from acute inflammation. SDMA levels are increased only in the course of CKD. Among the benefits of SDMA as a biomarker is the low level of intraindividual biological variability for dimethylarginine (5.8%) compared to cystatin C (8.6%) [9,17,18]. Due to the significance of arginine, citrulline, ADMA, and SDMA concentrations on the pathway of biochemical changes in the human body, these compounds were tested for their usefulness as potential biomarkers in the development of numerous disease entities [9,11,13,19,20].

The aim of the paper was to evaluate NO synthesis metabolites, i.e., asymmetric dimethylarginine (ADMA), symmetric dimethylarginine (SDMA), dimethylamine (DMA), arginine, citrulline in plasma of patients with different severity of chronic kidney disease, and to seek possible links between these parameters and the development of chronic kidney disease.

## 2. Materials and Methods

### 2.1. The Study Group

The study group included 48 patients with chronic kidney disease (16 girls and 32 boys) aged 3–18 years (median age 12 (IQR 6.75–16.0) years) treated in the Department of Pediatric Nephrology and Dialysis Station of the Wroclaw Medical University Hospital.

All the respondents met the inclusion criteria, which were:

- age of 3–18 years;
- diagnosed CKD of varying degrees of progression;
- and written consent to participate in the study.

Patients meeting the exclusion criteria were the failure to meet the inclusion criteria, recognition of another acute inflammatory disease, lack of cooperation and/or abnormalities that may affect the course of the research procedure were not eligible for the study. Taking GFR (mL/min/1.73 m^2^) values into account (estimated on the basis of Schwartz formula: eGFR (mL/min per 1.73 m^2^) = 0.413 * [height (cm)/serum creatinine (mg/dL)]), groups corresponding to a given stage of disease progression have been distinguished among CKD patients [21,22].

The size of groups in each stage of CKD is as follows:

**Group II**—15 patients with stage II CKD; including 11 boys, 4 girls;

**Group III**—16 patients with stage III CKD; including 10 boys, 6 girls;

**Group IV**—8 patients with stage IV CKD; including 4 boys, 4 girls;

**Group of children undergoing renal replacement therapy (RRT)**—9 patients undergoing RRT (hemodialysis, peritoneal dialysis); including 7 boys and 2 girls.

The cause of chronic kidney disease (CKD) in the studied population of patients was glomerulonephritis (18), pyelonephritis (20), and congenital defects of the urinary tracts (10). The hemodialysis [3–4 sessions per week (3–3.5 h)] was applied in 4 children using polysulfone membranes, NaHCO_3_-buffered dialysate, Ca^+2^ content—1.25 or 1.5 mmol/L. Peritoneal dialysis applied in 5 children includes NIPD—nocturnal intermittent peritoneal dialysis and CCPD—continuous cycling peritoneal dialysis with Baxter’s Home Choice using 1.36% or 2.27% Physioneal. The duration of renal replacement therapy (RRT) is 2.02 ± 0.51 years.

In the pediatric population with CKD, depending on the clinical condition and the results of laboratory tests, as well as the type of treatment of RRT in pharmacotherapy, the following drugs (in individual doses) were used: antihypertensive drugs (e.g., calcium channel blockers, angiotensin-converting enzyme inhibitors—ACEI inhibitors, β-blockers), vitamins: D3, C, B, folic acid, proton pump blockers, erythropoietin, calcium carbonate, iron preparations. 

Control group (group I)—33 healthy children (14 boys, 19 girls, median age 10 (7–14) years) with normal kidney function. These children were hospitalized in the Department of Pediatric Nephrology of the University Hospital due to suspected dysfunctions of the lower urinary tract (mainly nocturnal enuresis). On the basis of diagnostic tests carried out at that time, the above-mentioned abnormalities were excluded. None of these patients were diagnosed with a chronic disease or were not treated with specialist pharmacotherapy.

### 2.2. Ethical Issues

The research project has been approved by the Bioethics Committee of the Wroclaw Medical University, issue KB-369/2017 of 6 June 2017. All procedures involving human participants were in accordance with the highest ethical standards of the institutional research committee and were performed according to the Declaration of Helsinki on the treatment of human subjects and its later amendments. Both the children of all groups participating in the study (≥16 years) and their parents were informed about the aims, principles, benefits, and informed consent were obtained.

### 2.3. Collection of Test Material Samples

Blood samples from selected pediatric patients were collected in the morning in the sitting position, from the ulnar vein, during the planned diagnostic and therapeutic procedures. In dialysis patients, blood was collected between hemodialysis sessions. The material was collected after fasting. Blood was collected in test tubes with citrate anticoagulant and then the procedure regarding the material for the determination of arginine-related metabolites was followed as described in Section 3.2. The samples were stored in a low temperature freezer at −80 °C until the analysis of LC-MS/MS was performed.

**Figure 2:**
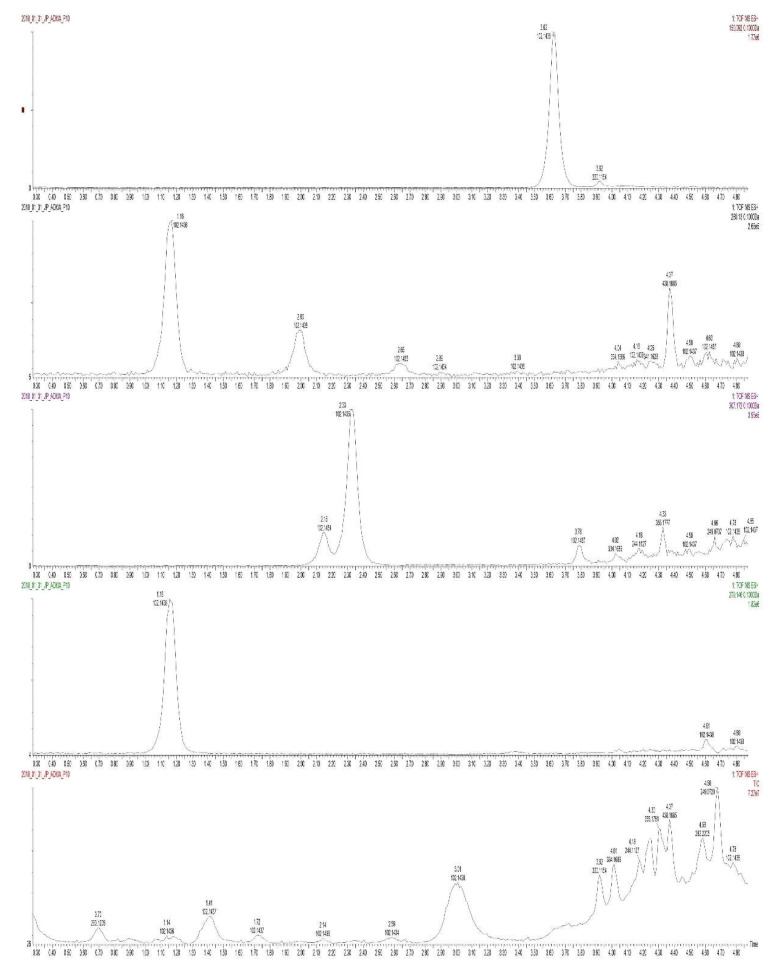
Extracted ion chromatograms for compounds derivatives (from top): DMA, citrulline, ADMA, SDMA, arginine, and total ionic chromatogram (TIC) of a representative patient sample.

Determination of NO synthesis-related metabolites was performed according to the developed method [23,24]. Arginine, ADMA, SDMA, citrulline, D6-DMA, benzoyl chloride (BCl), D0-DMA, and perchloric acid (PCA) were purchased from Sigma, while isotope-labelled arginine-HCl (D7, 98%) and ADMA (2,3,3,4,4,5,5-D7, 98%) were from Cambridge Isotope Laboratories. Briefly, 100 µL patient’s plasma sample was transferred to 2 mL Eppendorf tubes, then 10 µL of internal standard solution (50 µM D6-DMA; 20 µM D7-ADMA; 100 µM D7-arginine) and 50µL of borate buffer (0.025 M Na2B4O7-10H2O, 1.77 mM NaOH, pH 9.2) were added. Samples were shaken (1 min, 25 °C) and then 400 µL of acetonitrile and 10 µL of 10% BCl solution in acetonitrile were added. The solutions were shaken (5 min, 25 °C) and centrifuged (7 min, 15,000 RCF, 4 °C), then 100 μL of supernatant was transferred to glass vessels and diluted 4 times. Derivatives were analyzed using Acquity UHPLC liquid chromatograph with Acquity HSS T3 column (50 mm × 1.0 mm, 1.75 µm), a cooled autosampler, combined with Xevo G2 XS Q-TOF mass spectrometer (Waters), with an ESI-ion source operating at negative ionization mode. The data obtained were gathered and analyzed using Quanlynx software (version 4.0, Waters, Milford, MA, USA). The peak area ratio of the tested compound in relation to the peak area of the corresponding standard was used to perform linear regression analysis. A linear regression coefficient (r2) was calculated for each standard. For NO metabolites, it was above r2 > 0.993. A chromatogram of the representative patient sample and extracted ions chromatograms for individual compounds are shown on Figure 2.

### 2.4. Statistical Analysis

The results of the analyses were presented as mean (x), standard deviations (SD), median (M), lower and upper quartiles (25–75 Q). The equality of means in independent groups was tested with an ANOVA analysis of variance. A nonparametric Mann–Whitney U test and the Kruskal–Wallis test were performed. Spearman’s correlation coefficient was used to analyze the relationships between the tested parameters. Statistically significant differences at the *p* < 0.05 level were assumed. Statistical analysis was performed using STATISTICA 8 software (StatSoft, Tulsa, Poland).

## 3. Results

### 3.1. Clinical Characteristics

The blood biochemical parameters in the CKD children and control group are presented in Table 1. Detailed parameters for each CKD stage are presented in supplementary materials (Table S1).

### 3.2. Determination of NO Synthesis Metabolites and Comparison between the Study and Control Group

In most of the determined parameters, there are statistically significant differences between the group of CKD children and the control group. These differences occur in the case of the following metabolites: ADMA, SDMA, DMA, and citrulline (Table 2).

### 3.3. Comparison of the Results of Determinations with CKD Severity

**Figure 3:**
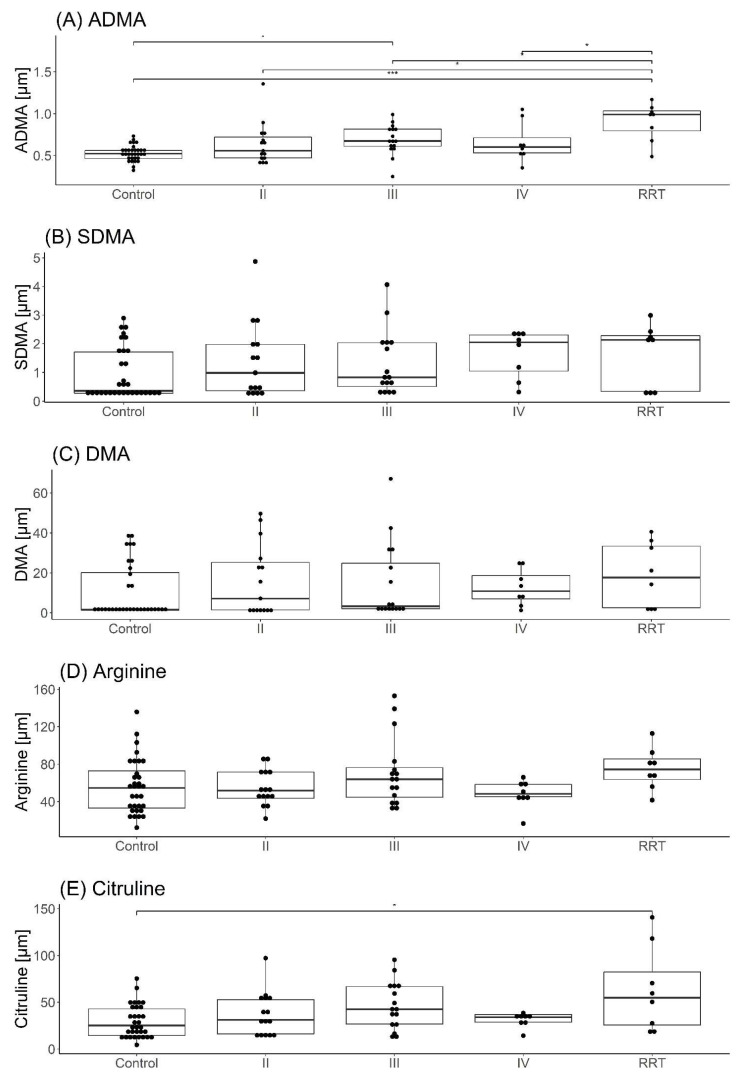
Comparison of differences in parameters depending on the severity of CKD stage (RRT—patients for renal replacement therapy, control—control group, II–IV stages of CKD progression). * *p* < 0.05, *** *p* < 0.001.

There were significant differences observed in levels of ADMA and Citrulline between groups within different stages of CKD (Kruskal–Wallis *p*-value ˂ 0.001 and 0.048, respectively) (Table 3, Figure 3). Post hoc comparisons showed that the RRT group had higher values of ADMA than other groups (RRT vs control *p* < 0.001, RRT vs II *p* = 0.01, RRT vs III *p* = 0.046, RRT vs IV *p* = 0.046) and the Control group had lower values of ADMA than III group. The Control group had lower values of citrulline than the RRT group *p* = 0.01.

### 3.4. Evaluation of Relationships in the Group of Children with CKD

**Figure 4:**
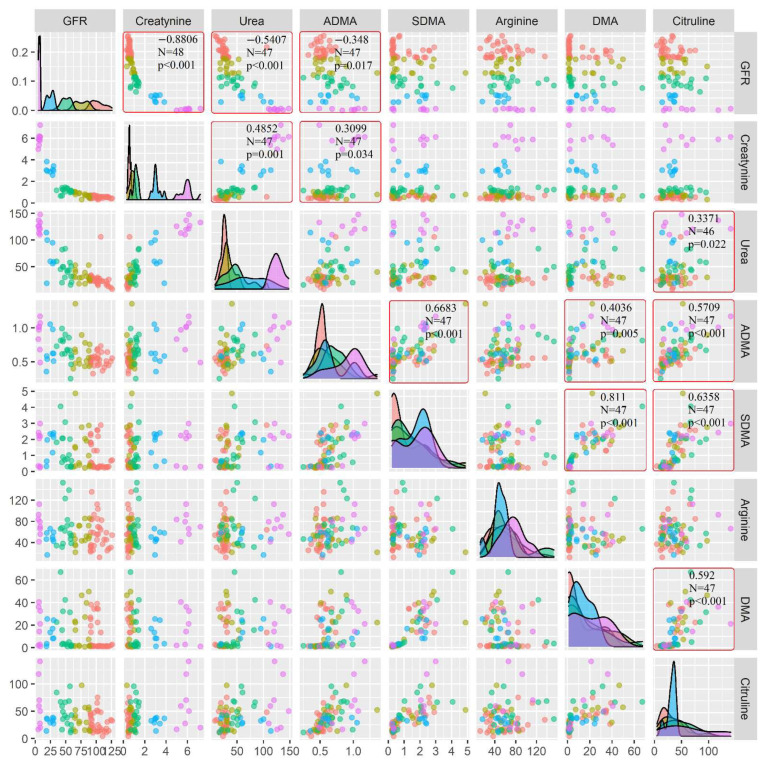
Correlations between studied NO synthesis metabolites. Legend: purple—healthy group, blue—Stage II CKD, green—stage III CKD, yellow—stage IV CKD, red—RRT. Density plots are shown diagonally. Spearman correlation coefficients with *p* values and number of patients are shown when statistically significant.

The results obtained in this work showed the correlations between studied NO synthesis metabolites in the group of children with CKD and are presented in Figure 4. A graphical presentation of the results of NO synthesis metabolites in groups of children with CKD is shown in Figure 3.

In the studied population of patients, positive correlations of statistical significance were found between:

- ADMA and creatinine, SDMA, DMA, and citrulline;
- SDMA and DMA, citrulline;
- DMA and citrulline;
- Citrulline and urea (Figure 4).

In the population of patients with CKD, negative correlations of statistical significance between ADMA and GFR were found (Figure 4).

## 4. Discussion

In recent years, the number of people suffering from civilization diseases, including CKD, has been systematically increasing. Therefore, early diagnosis of this disease and appropriate therapeutic strategies prevent its progression and the development of accompanying complications. In clinical practice, especially in the nephrological patient population, the size of endogenous creatinine clearance reflecting renal function is used to diagnose the severity of CKD, determine its progression, and monitor treatment [25]. Unfortunately, creatinine, urea, and microalbuminuria, as classic biomarkers used to assess kidney function, have disadvantages. Recent years of laboratory analyses have shown that compounds such as glycine, citrulline, ADMA, and SDMA can be potential biomarkers of CKD in patients regardless of the creatinine level in the study group [6,26,27,28,29].

In our analysis of metabolites related to the arginine-creatinine cycle, urea cycle, and arginine methylation metabolic pathways, statistically significant differences were found for ADMA (*p* < 0.001), SDMA (*p* = 0.018), DMA (*p* = 0.029) and citrulline (*p* = 0.012) between the CKD group and control (Table 2). It is so important because such statistical significance (in a similar but more numerous population of the studied children) was not found by El-Sadek et al. [11]. Similar results were found for the arginine value; our CKD children had lower levels (without statistical significance compared to the control) of arginine values in the CKD children were (statistically significantly lower) in El-Sadek et al. [11] (Table 2).

The occurrence of statistical differences for the studied parameters related to arginine may be directly related to the metabolism of NO. Chronic kidney disease is mentioned in the scientific literature as one of the examples of diseases associated with abnormal NO synthesis. Nitric oxide is believed to have anti-inflammatory, vasodilating, anti-adhesive, and anti-proliferative effects. Disturbances in all these processes are important for the development of disease late complications observed in the course of CKD [1,2]. ADMA is an endogenous inhibitor of nitric oxide synthase, and our child population with CKD was characterized by a high concentration of ADMA, which in turn disrupts the physiological NO (Table 2).

Despite the variety of published research results and conclusions drawn from them, most authors present results with increased concentrations of both dimethylarginins in CKD patients [6,11,30,31,32]. According to Boelaert et al., ADMA shows a very narrow range of normal concentrations; even a slight increase in its concentration may be associated with the risk of developing complications, e.g., cardiovascular [6]. The range of correct values we obtained for the control group confirmed this observation (Table 3). The results obtained for the ADMA and SDMA measurements in our study are also consistent with the reports of other authors who conducted the analysis in a similar patient population [6,11,30,31,32].

There were statistically significant differences in ADMA between the control group and patients treated with renal replacement therapy and between the control group and children with stage III CKD. This is partially confirmed by the results obtained by Snauwaert et al. in studies conducted on children with CKD treated conservatively. According to this Belgian research team, each uremic toxin they identify has a specific retention profile consistent with the corresponding stage of chronic kidney disease [31].

From a medical point of view, stage III CKD is the time of overt manifestation of clinical symptoms associated with this disease, and thus a statistically significant difference in ADMA between healthy and sick. ADMA levels in CKD children were statistically significantly higher in relation to particular stages of CKD (RRT vis II stage of CKD: *p* = 0.01; RRT vis III-IV stages of CKD: *p* < 0.046).

According to Shafi et al., ADMA and SDMA are pseudo-uremic toxins that can induce toxicity through a number of mechanisms, including disturbances in the synthesis of nitric oxide and the formation of reactive oxygen species [33]. Plasma ADMA levels do not correlate with blood creatinine or eGFR levels in children and adolescents with CKD, most likely because most ADMA is enzymatically degraded, in the opinion of Hsu Chien-Ning et al. [34]. While our observations showed a statistically negative correlation between ADMA and GFR in children with CKD (Figure 4), as also reported by Benito [26]. Therefore, we advocate Lim, who believes Caglara that circulating ADMA levels are elevated in patients with CKD, even before significant changes in GFR [35].

In the examined children with CKD, a statistically significant difference was found compared to the group of healthy subjects (*p* = 0.029) for DMA. The highest concentrations of this metabolite were reported in patients undergoing RRT compared to children from the control group as well as in relation to the remaining stages of the disease. DMA (like SDMA) is secreted with urine and accumulates in blood in case of kidney damage [36]. Unfortunately, currently little is known about the pathophysiological role of DMA. In the literature, there is no information concerning DMA analysis in patients with chronic kidney disease. Only individual works about this relationship have been published. One of the studies concerned the formation of active carcinogen—dimethylamine from DMA in the situation of elevated NO levels [37]. Patients with CKD with elevated ADMA also showed elevated DMA levels. The concentrations of ADMA and DMA are closely interrelated because DMA is formed by hydrolysis of ADMA. Despite research on the role of ADMA and SDMA in the development of CKD, there is no analysis of DMA as a factor that may correspond with the intensity of pathological lesions in kidneys. Due to the positive correlation between ADMA and DMA (r = 0.4036; *p* = 0.005) and between SDMA and DMA (r = 0.811; *p* < 0.001), further studies on the pathophysiological role of DMA should be carried out in patients with CKD in this paper (Figure 4). According to many researchers, conducting further studies that will fully explain the importance of metabolites associated with the arginine-creatinine cycle, urea cycle, and metabolic pathways of arginine methylation are justified [6,11,30,38].

The presence of statistically significant differences between the CKD patients and the control group for SDMA in this study confirms the role of this metabolite in the pathogenesis of renal lesions (*p* = 0.018). SDMA is mainly eliminated by the kidneys, therefore, there is a close correlation between plasma concentration of this analyte and kidney function. The results of the SDMA measurements of the analyzed group of patients are consistent with the reports of other authors. In the opinion of Kielstein et al., patients with ESRD undergoing dialysis showed the highest levels of SDMA [19], which is similar to our RRT pediatric patients.

According to scientific reports, SDMA is considered a valuable marker that is useful in the early diagnosis of chronic kidney disease. Additional benefits of SDMA as a marker of kidney disease are the lack of correlation between the serum concentration of this analyte and the influence of non-renal factors such as muscle mass, diet, inflammation, diabetes, or estrogen therapy [9,15,17,18]. According to Insa E Emrich and co-workers, SDMA predicts CKD progression and future atherosclerotic cardiovascular events more consistently than other methylarginines [1]. In the study by El-Sadek et al., regression analysis identified a high level of SDMA in the serum as a persistently significant predictor of low endogenous GFR [11].

Differences of statistical significance for plasma citrulline (*p* = 0.012) were also found between the control and CKD group. Citrulline concentration in the CKD patients was higher (mean 43.83 ± 28.31 µM) compared to the control group (mean 29.32 ± 17.29 µM) (Table 2). However, in CKD children, we found only statistically significant differences between the control and the RRT group (*p* = 0.01) (Table 3). Like DMA, citrulline is closely interrelated with ADMA because it is produced by hydrolysis of ADMA. However, the results of citrulline concentrations in pediatric patients with CKD differed from those obtained by other researchers [26,28,39,40]. Differences in the concentrations obtained may result from the use of different derivatization methods during sample preparation for LC-MS measurement.

The results obtained by Benito et al. show that simultaneous measurements of citrulline together with SDMA and S-adenosylmethionine can be useful in the diagnosis of CKD. The Benito research team found that the study of these three metabolites together with the level of creatinine in samples of patients with early-stage CKD allows increasing the probability of disease detection compared to the classification of patients based only on creatinine measurements [26]. Moreover, the measurement of these metabolites allowed a correct diagnosis for the remaining stages of CKD. Benito et al. also proposed to replace the Schwartz equation in pediatric patients with CKD and to develop a new equation based on the results of three above-mentioned metabolites and on commonly used parameters such as cystatin and creatinine concentrations, age, height, and weight. However, to replace the Schwartz equation in pediatric patients with CKD, further research is required on the larger population of study and control group [26]. The group of patients studied by Lim showed an increased level of citrulline in CKD patients [40]. Similar results were obtained by other research centers, and it was also noticed that an increased level of citrulline correlates particularly with the concentration of SDMA [26,28]. In our study, citrulline correlated statistically significantly positively with urea, ADMA, SDMA, and DMA (Figure 4).

In this study, no statistically significant differences were found in arginine concentrations between the control group (mean 56.25 ± 29.25 µM) and CKD patients (mean 62.65 ± 27.80 µM). Arginine concentration was higher in the population with CKD, especially in stage III of the disease and in children undergoing RRT. However, it seems interesting that the concentration of arginine in predialysis children (mean 48.16 ± 15.09) has drastically decreased even below the values of the control group (mean 56.26 ± 29.25). In the studies conducted by Benito’s team, lower arginine concentrations were found for CKD patients [26,28]. This metabolite was determined using the LC-QTOF-MS method [26,28]. There may be several factors that can affect such different results. In the case of arginine, the data presented in publications on the role of this metabolite in the pathomechanism of CKD are clear. One of the reasons for this is the use of different measurement techniques used for arginine tests. In addition, the use of different derivatization methods in the process of preparing samples for LC-MS measurement promotes discrepancies between the results presented in various publications [20,24,38]. For example, Rao and his research team used the method with the use of OPA for derivatization to simultaneously determine arginine, citrulline, and ADMA in plasma by reversed-phase high-performance liquid chromatography (HPLC) [20]. Attempts were also made to analyze OPA derivatives using a mass spectrometer. However, the results were characterized by relatively high LOD and LOQ values [41]. Derivatization with the use of OPA and then HPLC measurements using a fluorescence detector were used for tests by Chen et al. [38]. The use of various derivatization reagents and a relatively low specific detector could lead to higher results in Chen et al. compared to the results of this study.

By analyzing the available scientific literature, it is possible to formulate a hypothesis regarding the relationship between nucleotide metabolism and arginine metabolites [42,43]. All NOS (Nitric oxide synthases) isoforms use arginine and molecular oxide as substrates and require cofactors such as, e.g., reduced NADPH. Fliser explains the increased ADMA concentration in CKD patients due to inhibition of NO synthesis [44]. On the other hand, SDMA has a weak inhibitory effect on NOS, but may indirectly interfere with NO synthesis by competing with arginine for membrane transport. SDMA is considered a good marker for determining kidney function [19].

In our results, both ADMA and SDMA show a negative correlation with eGFR, but in the case of ADMA, it is statistically significant, while for SDMA, it is not so significant. There is also plausibility of type II error after taking into account a relatively small sample group. ADMA metabolism by DDAH 1 and 2 isoforms is the major route of ADMA clearance from the circulation, changes in DDAH expression levels, and/or activity are a possible mechanisms leading to changes in the concentration of ADMA.

On the other hand, existence cannot be ruled out of stereo-specific transporters dedicated selectively to ADMA and for SDMA.

It is possible that alternative metabolic paths as well cytological transporters are activated when renal function is impaired. Those alternative paths of metabolism of ADMA and SDMA may be more effective for SDMA, therefore, there is an increased excretion of SDMA in the urine.

However, our hypothesis assumes that the physiological factor should be sought, and not the biochemical parameters, for the relationship/correlation of ADMA vis GFR and SDMA vis GFR, as the very definition of GFR allows this to pass.

The results obtained in this study shed more light on the complicated pathomechanism of metabolic profiles in chronic kidney disease. We can agree with the opinion of Benito et al. that the multivariate analysis of data on the plasma concentration of urea cycle metabolites, arginine methylation, and the metabolic pathways of arginine and creatinine in pediatrics makes it possible to “classify” a child to a specific stage of the disease with accuracy 74%, with up to 90% of the current misdiagnosis of CKD progression (by a degree above or below) by a physician [26]. Therefore, further metabolic studies are necessary, which will undoubtedly contribute to the identification of pathognomonic biomarkers of CKD in the near future.

## 5. Limitations

Considering the overall design of our observation, it should be emphasized that our observations have limitations. It would be necessary to analyze more patients in each stage of CKD. Increasing the number of children could sharpen the results towards statistically significant differences. It would also be interesting to expand the research group to include children after kidney transplantation. Another limitation of the study is the age characteristics of the patients, as the literature data show different values of ADMA concentration depending on the age of the pediatric population [45]. Pharmacotherapy is also important and some drugs can modify ADMA levels. Therefore, the solution to these doubts, and at the same time preserving the benefits of the study group (e.g., lack of comorbidities, multi-drug pharmacotherapy, etc.), would be to deepen the research in the group of patients after adolescence, i.e., adolescent adults.

## 6. Conclusions

1. Children with CKD develop disturbances in most metabolites of NO synthesis.
2. These disorders worsen with the progression of CKD.
3. Dialysis children treated show the greatest disturbances of plasma ADMA and citrulline levels.
4. ADMA seems to be a good indicator of the gradual progression of the CKD, which is proved by the negative correlation with eGFR.
5. NO metabolites can possibly be promising markers of CKD stage and severity. Future research is needed to establish their clinical potential.

## Figures and Tables

**Table 1:** The biochemical characteristics of the blood of the studied patient population and control group.

Studied Parameter	Study Patient Group		Control Group		
	**N**	Median (IQR)	N	Median (IQR)	*p*-value
GFR [mL/min/1.73 mpc]	48	48.69 (23.36–65.38)	33	102.12 (92.93–110.13)	<0.001
creatinine (mg/dL)	48	1.18 (0.92–3.06)	33	0.55 (0.5–0.61)	<0.001
inorganic phosphorus (mg/dL)	44	4.9 (4.35–5.5)	33	5.1 (4.6–6.1)	0.142
calcium (mg/dL)	43	9.8 (9.6–10.1)	32	10 (9.88–10.4)	0.020
sodium (mg/dL)	48	139 (137–142)	33	138 (137–140)	0.376
potassium (mg/dL)	48	4.42 (4.19–4.66)	32	4.3 (4.2–4.62)	0.644
urea (mg/dL)	47	50 (30–89.5)	32	24.5 (21.25–28.25)	<0.001
Hb (g/dL)	48	11.7 ± 1.51	33	14.4 ± 1.74	<0.001
Ht (%)	48	34.33 ± 4.15	33	41.38 ± 4.88	<0.001
RBC [mln/mm^3^]	48	4.14 ± 0.71	33	4.85 ± 0.55	<0.001

**Table 2:** Comparison of test results for both study and control group (N = 79, data was missing for 2 patients). A comparison of parameter results was performed using the Mann–Whitney U test.

Studied Parameter	Study Group/CKD Group	Control Group	
**N**	47	32	
	Median (IQR)	Median (IQR)	*p*-value
ADMA [µM]	0.66 (0.53–0.84)	0.52 (0.47–0.56)	<0.001
SDMA [µM]	1.18 (0.38–2.17)	0.36 (0.28–1.71)	0.018
Arginine [µM]	57.04 (44.96–71.82)	54.72 (33.1–72.91)	0.230
DMA [µM]	8.08 (1.74–24.86)	1.65 (1.28–20.15)	0.029
Citrulline [µM]	37.01 (26.23–56.93)	25.27 (14.38–42.89)	0.012

**Table 3:** Values of measured NO synthesis metabolites parameters for individuals with different stages of CKD and Control group. *p*-value is for the difference in distribution between groups. A comparison was performed using Kruskal–Wallis test.

	Control	II	III	IV	RRT	*p*-Value
**ADMA [µM]**	0.52 (0.47–0.56)	0.56 (0.47–0.72)	0.67 (0.61–0.82)	0.6 (0.53–0.71)	0.99 (0.8–1.03)	<0.001
**SDMA [µM]**	0.36 (0.28–1.71)	0.99 (0.37–1.98)	0.84 (0.51–2.03)	2.05 (1.05–2.3)	2.13 (0.34–2.28)	0.14
**Arginine [µM]**	54.72 (33.1–72.91)	51.82 (43.59–71.66)	63.98 (44.64–76.33)	48.36 (45.19–58.37)	74.54 (63.54–85.74)	0.151
**DMA [µM]**	1.65 (1.28–20.15)	7.21 (1.46–25.27)	3.36 (2.11–24.84)	10.88 (6.94–18.75)	17.73 (2.55–33.43)	0.263
**Citrulline [µM]**	25.27 (14.38–42.89)	31.16 (16.22–52.77)	42.57 (26.77–66.72)	34.12 (28.74–36.92)	54.96 (25.71–82.3)	0.048
**GFR [** **mL/min/1.73 mpc]**	102.12 (92.93–110.13)	76.14 (65.58–85.64)	50.53 (45.04–57.13)	26.48 (19.3–29.08)	6.58 (6.03–7.94)	<0.001
**Creatinine [** **mg/dL]**	0.55 (0.5–0.61)	0.8 (0.69–0.96)	1.17 (1.04–1.27)	3.01 (2.94–3.18)	5.89 (5.78–6.13)	<0.001
**Urea [** **mg/dL]**	24.5 (21.25–28.25)	30 (27.5–39.5)	44 (31.5–52.75)	60 (57.5–97.5)	126 (120–133)	<0.001

## Supplementary Materials

The following supporting information can be downloaded at: https://www.mdpi.com/article/10.3390/cimb44080242/s1, Table S1: Clinical and biochemical characteristics of patient groups.
