# Supplementary material for: Nitric Oxide Synthesis Metabolites—As Potential Markers in Chronic Kidney Disease in Children
Source: Curr Issues Mol Biol. 2022 Aug 7;44(8):3518–32. doi: 10.3390/cimb44080242 (PMC9406431; doi:10.3390/cimb44080242)
Supplement: Supplementary file 1 [file cimb-44-00242-s001.zip › cimb-1808490-supplementary.pdf]

Table S1. Clinical and biochemical characteristics of patient groups.

Studied parameter	Control group	II	III	IV	RRT
Age	10 (7.25 - 13.75)	11 (10 - 14.25)	12 (10.5 - 15.25)	8.5 (3 - 14.25)	15 (8 - 17.75)
GFR [ml/min/1.73mpc]	101.7 (92.49 - 109.72)	74.25 (65.57 - 83.15)	50.53 (45.04 - 57.13)	26.48 (19.3 - 29.08)	5 (5 - 5)
creatinine (mg/dl)	0.58 (0.5 - 0.71)	0.81 (0.67 - 0.98)	1.16 (0.99 - 1.23)	1.99 (1.17 - 2.34)	1.63 (1.2 - 2.55)
inorganic phosphorus (mg/dl)	5.1 (4.6 - 6.1)	4.9 (4.6 - 5.5)	5 (4.47 - 5.62)	5.2 (4.75 - 5.75)	4.45 (3.95 - 4.8)
calcium (mg/dl)	10 (9.88 - 10.4)	10 (9.8 - 10.2)	9.75 (9.6 - .88)	9.8 (9.05 - 9.9)	9.8(9.38-0.12)
sodium (mg/dl)	138 (137 - 140)	139 (137 - 139.75)	141(138-142.25)	141 (137 - 143.25)	138.5(137-140)
potassium (mg/dl)	4.31 (4.2 - 4.6)	4.3 (4.1 - 4.62)	4.63 (4.34 - 4.68)	4.55 (4.35 - 4.75)	4.22(4.09- 4.36)
urea (mg/dl)	24 (22 - 28)	31 (29 - 39.75)	44 (31.5 - 52.75)	56 (14.55 - 79.5)	55(34.75- 6.75)
Hb (g/dl)	14.47 ± 1.72	13.03 ± 0.99	12.03 ± 1.26	10.37 ± 0.64	10.5 ± 1.03
Ht (%)	41.38 ± 4.88	36.06 ± 5.18	35.54 ± 3.14	31.69 ± 2.17	32.09 ± 3.46
RBC [mln/mm^3]	4.87 ± 0.55	4.99 ± 0.45	4.12 ± 0.36	3.53 ± 0.26	3.49 ± 0.34
